# Pain distribution and functional outcomes after lateral unicompartmental versus total knee arthroplasty for isolated lateral compartment osteoarthritis with anterior knee pain

**DOI:** 10.3389/fmed.2026.1790404

**Published:** 2026-02-20

**Authors:** Zhenbao Lu, Xiaodan Lin, Jianfu Zhu, Xu Wang, Xiaohong Fan, Jiliang Chen, Qiujin Xia, Chengshou Lin, Qingshan Xu, Qijin Wang

**Affiliations:** 1 Department of Orthopaedics, Affiliated Mindong Hospital, Fujian Medical University, Ningde, Fujian, China; 2 Department of Neurology, Affiliated Mindong Hospital, Fujian Medical University, Ningde, Fujian, China; 3 School of New Energy and Intelligent Manufacturing, Ningde Vocational and Technical College, Ningde, Fujian, China

**Keywords:** anterior knee pain, lateral unicompartmental knee arthroplasty, lateral compartment osteoarthritis, pain distribution, patellofemoral joint, patient-reported outcome measures, precision medicine, total knee arthroplasty

## Abstract

**Background:**

Isolated lateral compartment osteoarthritis (LCOA) with anterior knee pain (AKP) represents a lateral tibiofemoral–patellofemoral phenotype. Evidence comparing lateral unicompartmental knee arthroplasty (LUKA) and total knee arthroplasty (TKA) in this subgroup is limited for compartment-specific pain. This study compared pain distribution and function when both procedures were performed within a lateral parapatellar pathway.

**Methods:**

This retrospective cohort included 115 patients with isolated LCOA and AKP who underwent LUKA (*n* = 52) or TKA (*n* = 63) and completed 24 months of follow-up. All cases used a lateral parapatellar approach with patellar denervation and indication-based lateral retinacular release. The primary outcome was the Knee Society Score (KSS) at 24 months. Secondary outcomes were WOMAC, exploratory visual analogue scale (VAS) scores for lateral tibiofemoral (LC-VAS) and patellofemoral pain (PFC-VAS) at 3 and 24 months, perioperative metrics, complications, and reoperation-free survival. Group comparisons used unadjusted tests. For 24-month KSS and WOMAC, exploratory multivariable linear regression was complemented by propensity score weighting [stabilised inverse probability of treatment weighting (IPTW) and overlap weighting, with balance assessed using standardised mean differences (SMD)].

**Results:**

Baseline characteristics and preoperative scores were comparable. LUKA was associated with shorter operative time, less blood loss, and shorter hospital stay. At 3 months, KSS was higher and WOMAC lower after LUKA, with lower LC-VAS and similar PFC-VAS. At 24 months, no statistically significant between-group differences were detected in KSS, WOMAC, LC-VAS, and PFC-VAS. Regression estimates were small (KSS adjusted mean difference 0.51, 95% confidence interval (CI) −0.58 to 1.60, and WOMAC −0.62, 95% CI −1.85 to 0.62). Propensity score–weighted estimates were consistent, and 95% CIs remained within published minimal clinically important difference (MCID) thresholds for KSS (5 points) and WOMAC total (10 points). No revisions occurred after LUKA. One infection treated with debridement, antibiotics, and implant retention occurred after TKA.

**Conclusion:**

Within this standardised pathway, LUKA enabled faster early recovery and lower lateral pain with less surgical burden, while no statistically significant between-group differences were detected in 24-month pain distribution or function. The data primarily describe early recovery trajectories and pain distribution in this phenotype and warrant multicentre confirmation.

## 1 Introduction

Knee osteoarthritis (KOA) is a major cause of pain and disability in older adults, characterised by progressive cartilage degeneration, subchondral bone remodelling, and persistent joint symptoms (1). As populations age, the prevalence of KOA and its associated healthcare burden continue to increase worldwide (2, 3). Isolated lateral compartment osteoarthritis (LCOA), a less common subtype, accounts for approximately 10–15% of symptomatic cases and is often associated with valgus malalignment and lateral compartment overload (4–7).

Anterior knee pain (AKP) and patellofemoral osteoarthritis (PFOA) are frequent in KOA and contribute disproportionately to symptoms, functional limitation, and postoperative dissatisfaction. In LCOA, some patients also have PFOA, forming a lateral tibiofemoral–patellofemoral phenotype characterised by valgus alignment, lateral compartment overload, and AKP (4). This combined pattern complicates decision-making because procedure selection and patellofemoral management remain contentious, and AKP may reflect multiple, overlapping mechanisms beyond radiographic degeneration (8).

For patients with advanced disease who do not respond to nonoperative treatment, surgical reconstruction is often required. Total knee arthroplasty (TKA) addresses multicompartmental involvement, but it may be more than is needed for truly isolated LCOA because it involves wider bone resection and a longer rehabilitation course (9). By contrast, lateral unicompartmental knee arthroplasty (LUKA) preserves bone and is associated with shorter operative time and faster early functional recovery (10, 11). However, evidence comparing unicompartmental knee arthroplasty (UKA) with TKA, and evidence on the influence of PFOA or AKP, largely comes from medial UKA cohorts (12). Those studies suggest that mild-to-moderate PFOA or AKP does not inevitably compromise function or survivorship after medial UKA, yet their relevance to LCOA with coexisting AKP or PFOA remains uncertain. Data specifically addressing LUKA in this lateral tibiofemoral–patellofemoral phenotype are limited. Although recent series and meta-analyses in isolated LCOA report encouraging survivorship and function and suggest potential advantages over TKA, many cohorts exclude symptomatic patellofemoral disease or apply non-standardised, surgeon-dependent patellofemoral management (13–15).

Pain distribution in isolated LCOA with coexisting AKP is seldom reported in LUKA–TKA comparisons, and patellofemoral co-interventions remain variable across studies. Building on pain-mapping approaches used in KOA and arthroplasty populations (16–18) and on the increasing use of digital pain manikins to capture anatomic pain location patterns (18, 19), we conducted a single-centre retrospective cohort study in which LUKA and TKA were compared within a uniform lateral parapatellar pathway, including routine patellar denervation and lateral retinacular release. Outcomes were evaluated at 3 and 24 months using KSS, WOMAC, and two compartment-specific visual analogue scale (VAS) ratings for lateral tibiofemoral and patellofemoral pain (LC-VAS and PFC-VAS). LC-VAS and PFC-VAS were prespecified as exploratory descriptors of pain distribution rather than as validated compartment-specific instruments. We expected LUKA to yield faster early recovery and lower perioperative burden, whereas between-group differences in 24-month function and pain distribution would be small.

## 2 Materials and methods

### 2.1 Study design and ethical considerations

This single-centre retrospective cohort study was performed at a tertiary hospital. The protocol was approved by the Ethics Committee of the Affiliated Mindong Hospital, Fujian Medical University (Fujian, China; approval No. H2025010301). All patients provided written consent at the time of surgery for the future use of anonymised clinical and radiographic data. This analysis used anonymised data only and required no additional study-specific consent. The study was conducted in accordance with the Declaration of Helsinki and reported in line with the STROBE (Strengthening the Reporting of Observational Studies in Epidemiology) guidance for cohort studies. No animal experiments were involved.

### 2.2 Patient selection

This retrospective study included consecutive patients with isolated LCOA and coexisting AKP who underwent primary knee arthroplasty at our institution between January 2018 and November 2023. Patients were identified from the institutional arthroplasty registry and verified using outpatient records and preoperative imaging. Patients with follow-up shorter than 24 months or incomplete key outcome data were excluded.

Inclusion criteria were: age ≥ 40 years; correctable valgus deformity with valgus alignment ≤ 10°; knee flexion ≥ 100° and flexion contracture ≤ 10°; radiographic LCOA with Kellgren–Lawrence (KL) grade III–IV in the lateral compartment and preserved medial compartment joint space (KL grade 0–II); and mild-to-moderate patellofemoral compartment degeneration (KL grade I–II). AKP was defined as patient-reported pain around or behind the patella during activities such as stair climbing, rising from a chair, or prolonged sitting, supported by clinical assessment.

Exclusion criteria were advanced patellofemoral osteoarthritis (KL grade III–IV), inflammatory arthritis, major coronal plane instability (medial or lateral collateral ligament insufficiency), severe bone loss of the lateral tibial plateau, and loss to follow-up before 24 months. In addition, implant-specific eligibility considerations informed selection of LUKA versus TKA, including ligament integrity, deformity correctability, and the extent of lateral tibial bone loss.

Treatment allocation was non-randomised and reflected routine clinical decision-making documented in the medical records. Procedure choice was based on preoperative clinical and radiographic assessment of compartment involvement, deformity correctability, ligament integrity, and lateral tibial bone loss. LUKA was generally selected when isolated lateral disease with a preserved medial compartment and adequate ligament stability was present and the deformity was clinically correctable. TKA was selected when these implant-specific conditions were not met or when both options were considered feasible but the patient preferred TKA after counselling.

### 2.3 Surgical procedures

All procedures were performed by a single senior orthopaedic surgeon using a standardised lateral parapatellar approach with a layered, Z-shaped capsulotomy. In our institution, this constituted a unified lateral parapatellar pathway applied to both LUKA and TKA to standardise exposure, patellofemoral assessment, and perioperative management across implant types. Compared with conventional medial parapatellar workflows, the lateral arthrotomy provides direct lateral access for balancing and facilitates intraoperative patellar tracking assessment, thereby reducing variability in patellofemoral handling between groups. Patellar denervation was achieved by circumferential electrocautery along the patellar rim (continuous rim cauterisation along the patellar margin), followed by lateral retinacular release in a graded manner when indicated by persistent lateral tilt or subluxation on intraoperative tracking assessment to facilitate patellofemoral tracking. Patellar tracking was assessed after trial implantation through passive knee flexion–extension and recorded as satisfactory/unsatisfactory in the operative note. The patella was not resurfaced in any case. In LUKA, bone resections were performed using conventional instrumentation, and a cemented fixed-bearing unicompartmental implant was inserted. In TKA, femoral and tibial resections were performed using intramedullary and extramedullary alignment guides, respectively, followed by implantation of a cemented fixed-bearing total knee prosthesis; a cruciate-retaining or posterior-stabilised design was selected according to posterior cruciate ligament integrity.

All patients received perioperative antibiotic prophylaxis, standardised thromboprophylaxis, and postoperative multimodal analgesia according to institutional protocols. Rehabilitation began on postoperative day 1 and followed the same programme in both groups.

### 2.4 Data collection and outcome measures

Collected variables included demographics (age, sex, body mass index [BMI]), perioperative parameters (operative time, incision length, and intraoperative blood loss), length of hospital stay, complications and reoperations, and clinical outcomes assessed preoperatively and at 3 and 24 months. Intraoperative blood loss was extracted from the operative record as estimated blood loss (EBL); no formula-based derivation was applied. Functional outcomes were evaluated using the Knee Society Score (KSS) (20) and the Western Ontario and McMaster Universities Osteoarthritis Index (WOMAC) (21). The prespecified primary outcome was the KSS at 24 months. Key secondary outcomes were the WOMAC at 24 months and compartment-specific pain ratings (LC-VAS and PFC-VAS) at 24 months. Exploratory outcomes included KSS, WOMAC, LC-VAS, and PFC-VAS at 3 months, perioperative parameters (operative time, incision length, intraoperative blood loss, and length of hospital stay), complications and reoperation-free survival (any reoperation on the index knee as an event).

Pain was measured using a 0–10 VAS (22), where 0 indicated no pain and 10 indicated the worst imaginable pain. At each assessment, patients were asked to rate their average pain during usual daily activities over the preceding week (i.e., activity-related pain rather than pain at rest, typically during weight-bearing ambulation and stair negotiation). All LC-VAS and PFC-VAS ratings were obtained face-to-face during routine outpatient follow-up visits and were recorded by a trained assessor using scripted, standardised instructions. Patients rated pain predominantly perceived in the lateral tibiofemoral compartment (LC-VAS) and around or behind the patella (PFC-VAS) separately, using standardised anatomical diagrams and uniform verbal instructions. During the physical examination, the assessor indicated the corresponding anatomical areas directly on the patient’s knee (lateral joint line/lateral tibiofemoral region and the peripatellar region) and asked the patient to describe and rate pain attributable to each indicated location separately (0–10 VAS). This anatomy-guided localisation approach was adapted from knee pain mapping and digital pain manikin methodologies that have been used to characterise pain location patterns in KOA and after knee arthroplasty (16–18). Because LC-VAS and PFC-VAS have not undergone formal psychometric validation for compartment-specific discrimination, they were prespecified as exploratory endpoints to describe patient-reported pain distribution and were interpreted accordingly. Between-group comparisons for these outcomes were based on the unadjusted analyses described below.

Postoperative radiographs were obtained as part of routine care; however, quantitative radiographic assessments (e.g., limb alignment, component coronal alignment/varus–valgus, tibial slope, and implant positioning) were not systematically measured and were therefore not included in the predefined outcomes. Reoperation-free survival was summarised using Kaplan–Meier curves. Any reoperation on the index knee was treated as an event. Given the low event rate and the fixed follow-up window, survivorship analyses were interpreted descriptively (through 24 months).

### 2.5 Statistical analysis

All analyses were performed using IBM SPSS Statistics (version 26.0; IBM Corp., Armonk, NY, United States) and R (version 4.3.2; R Foundation for Statistical Computing, Vienna, Austria). Continuous variables were compared using independent-samples t tests (Welch’s *t* test when appropriate) or the Mann–Whitney U test, and categorical variables using the chi-square or Fisher’s exact test. For the prespecified primary outcome (24-month KSS) and key secondary outcome (24-month WOMAC), exploratory multivariable linear regression was performed with treatment group as the main predictor, adjusting for age, sex, BMI, ASA class, operated side, and the corresponding preoperative score.

To assess robustness to measured confounding, propensity score–weighted sensitivity analyses (stabilised inverse probability of treatment weighting [IPTW] and overlap weighting) were conducted as robustness checks alongside the primary analyses, recognising that these methods address measured covariates only. Propensity scores were estimated using logistic regression with the same prespecified covariates, and covariate balance was evaluated using absolute standardised mean differences (<0.10). Treatment effects were reported as mean differences with 95% confidence intervals from weighted linear regression with robust (sandwich) standard errors. Reoperation-free survival was summarised descriptively using Kaplan–Meier curves given the low event rate. All tests were two-sided, and *p* < 0.05 was considered statistically significant.

As this was a retrospective cohort with a fixed study period, no *a priori* sample size calculation was performed. Accordingly, inference emphasised effect estimation with 95% confidence intervals, and no equivalence or non-inferiority framework was prespecified.

## 3 Results

### 3.1 Baseline characteristics

After exclusions (two patients with incomplete records, one lost to follow-up, and one who declined research use of their data), 115 patients were included in the final analysis: 52 underwent LUKA and 63 underwent TKA. Baseline characteristics were broadly comparable between groups, including age, sex, body mass index (BMI), ASA class, operated side, and Kellgren–Lawrence grades in the lateral compartment and patellofemoral compartment. Baseline pain and function scores (LC-VAS, PFC-VAS, KSS, and WOMAC) were also similar between groups. Baseline variables are summarised in Table 1.

**Table 1:** Baseline demographic and clinical characteristics of patients undergoing LUKA or TKA.

Parameters	LUKA (*n* = 52)	TKA (*n* = 63)	*P* value
Gender			
Female	28 (53.8%)	37 (58.7%)	0.599^b^
Male	24 (46.2%)	26 (41.3%)	
Age (years)	68.0 ± 9.5	67.3 ± 7.2	0.658^d^
BMI (kg/m^2^)	25.51 ± 1.64	25.75 ± 1.39	0.399^a^
Operation side			
Left	20 (38.5%)	23 (36.5%)	0.829^b^
Right	32 (61.5%)	40 (63.5%)	
ASA			
1–2	39 (75.0%)	38 (60.3%)	0.095^b^
3–4	13 (25.0%)	25 (39.7%)	
OA grade in LC (KL)			
3	32 (61.5%)	33 (52.4%)	0.324^b^
4	20 (38.5%)	30 (47.6%)	
OA grade in PFC (KL)			
1	21 (40.4%)	28 (44.4%)	0.661^b^
2	31 (59.6%)	35 (55.6%)	
LC-VAS	5.0 (4.0–6.0)	5.0 (5.0–6.0)	0.161^c^
PFC-VAS	2.0 (2.0–3.0)	2.0 (2.0–3.0)	0.871^c^
KSS	61.4 ± 3.5	60.2 ± 3.7	0.077^a^
WOMAC	64.5 (62.8–66.3)	65.0 (63.0–67.0)	0.672^c^

a *P* values were calculated using the independent samples *t*-test.

b Fisher’s exact test.

c Mann–Whitney U test.

d Welch *t*-test.

Values are presented as mean ± SD or median (IQR) according to distribution.

LUKA, lateral unicompartmental knee arthroplasty; TKA, total knee arthroplasty; BMI, body mass index; ASA, American Society of Anesthesiologists; KL, Kellgren–Lawrence classification; LC, lateral compartment; PFC, patellofemoral compartment; VAS, visual analogue scale; KSS, Knee Society Score; WOMAC, Western Ontario and McMaster Universities Osteoarthritis Index; IQR, interquartile range.

### 3.2 Perioperative comparison

Patients undergoing LUKA had a significantly shorter operative time than those undergoing TKA (54.38 ± 4.58 vs. 71.22 ± 5.14 min, *p* < 0.001). In addition, LUKA was associated with lower intraoperative blood loss (58.50 [54.00–70.00] vs. 208.00 [180.00–239.00] mL, *p* < 0.001), a shorter incision length (8.55 [8.10–9.80] vs. 14.20 [13.45–15.10] cm, *p* < 0.001), and a shorter hospital stay (5.00 [4.00–5.00] vs. 7.00 [6.00–8.00] days, *p* < 0.001) (Table 2).

**Table 2:** Perioperative surgical parameters in patients undergoing LUKA or TKA.

Parameters	LUKA (*n* = 52)	TKA (*n* = 63)	*P* value
Operative time (min)	54.38 ± 4.58	71.22 ± 5.14	<0.001^a^
Estimated blood loss (mL)	58.50 (54.00–70.00)	208.00 (180.00–239.00)	<0.001^b^
Incision length (cm)	8.55 (8.10–9.80)	14.20 (13.45–15.10)	<0.001^b^
Hospital stay (days)	5.00 (4.00–5.00)	7.00 (6.00–8.00)	<0.001^b^

a *P* values were calculated using the independent samples t-test.

b Mann–Whitney U test.

Values are presented as mean ± SD or median (IQR) according to distribution.

LUKA, lateral unicompartmental knee arthroplasty; TKA, total knee arthroplasty; IQR, interquartile range.

### 3.3 Postoperative outcomes

At 3 months, LUKA was associated with better function than TKA, with higher KSS and lower WOMAC, and lower lateral compartment pain (LC-VAS), while patellofemoral pain (PFC-VAS) did not differ significantly (Table 3). By 24 months, KSS, WOMAC, LC-VAS, and PFC-VAS did not differ significantly between groups (Table 3). In exploratory multivariable linear regression adjusting for age, sex, body mass index, ASA class, operated side, and the corresponding preoperative score, the adjusted mean difference in 24-month KSS was 0.51 points (95% confidence interval [CI] −0.58 to 1.60; *p* = 0.357) and the adjusted mean difference in total WOMAC was −0.62 points (95% CI −1.85 to 0.62; *p* = 0.326). Propensity score–weighted sensitivity analyses were consistent with the main models. Stabilised IPTW achieved good covariate balance (maximum absolute standardised mean difference = 0.058; maximum stabilised weight = 2.63), yielding a 24-month KSS mean difference of 0.46 (95% CI −0.55 to 1.47) and a WOMAC mean difference of −0.72 (95% CI −1.92 to 0.48). Overlap weighting produced similar estimates (KSS 0.36, 95% CI −0.66 to 1.37; WOMAC −0.63, 95% CI −1.84 to 0.57). At 24 months, LC-VAS and PFC-VAS values were close to the lower bound and KSS values were high, suggesting potential floor and ceiling effects that may reduce the ability to detect small residual between-group differences.

**Table 3:** Postoperative functional outcomes and compartment-specific pain at 3 and 24 months in patients undergoing LUKA or TKA.

Parameters	LUKA (*n* = 52)	TKA (*n* = 63)	*P* value
3-month KSS	81.0 (78.8–82.3)	76.0 (73.0–83.0)	<0.001^b^
3-month WOMAC	47.5 (45.0–50.0)	52.0 (49.0–55.5)	<0.001^b^
3-month LC-VAS	2.0 (2.0–2.3)	3.0 (2.0–4.0)	<0.001^b^
3-month PFC-VAS	1.0 (1.0–2.0)	1.0 (1.0–2.0)	0.639^b^
24-month KSS	92.44 ± 2.93	91.95 ± 2.65	0.349^a^
24-month WOMAC	16.60 ± 3.37	17.13 ± 3.10	0.382^a^
24-month LC-VAS	1.0 (1.0–2.0)	2.0 (1.0–2.0)	0.237^b^
24-month PFC-VAS	1.0 (0–1.0)	1.0 (0–2.0)	0.143^b^

a *P* values were calculated using the independent samples *t*-test.

b Mann–Whitney U test.

Values are presented as mean ± SD or median (IQR) according to distribution.

LUKA, lateral unicompartmental knee arthroplasty; TKA, total knee arthroplasty; KSS, Knee Society Score; WOMAC, Western Ontario and McMaster Universities Osteoarthritis Index; LC, lateral compartment; PFC, patellofemoral compartment; VAS, visual analogue scale; IQR, interquartile range.

### 3.4 Reoperation-free survival

**Figure 1:**
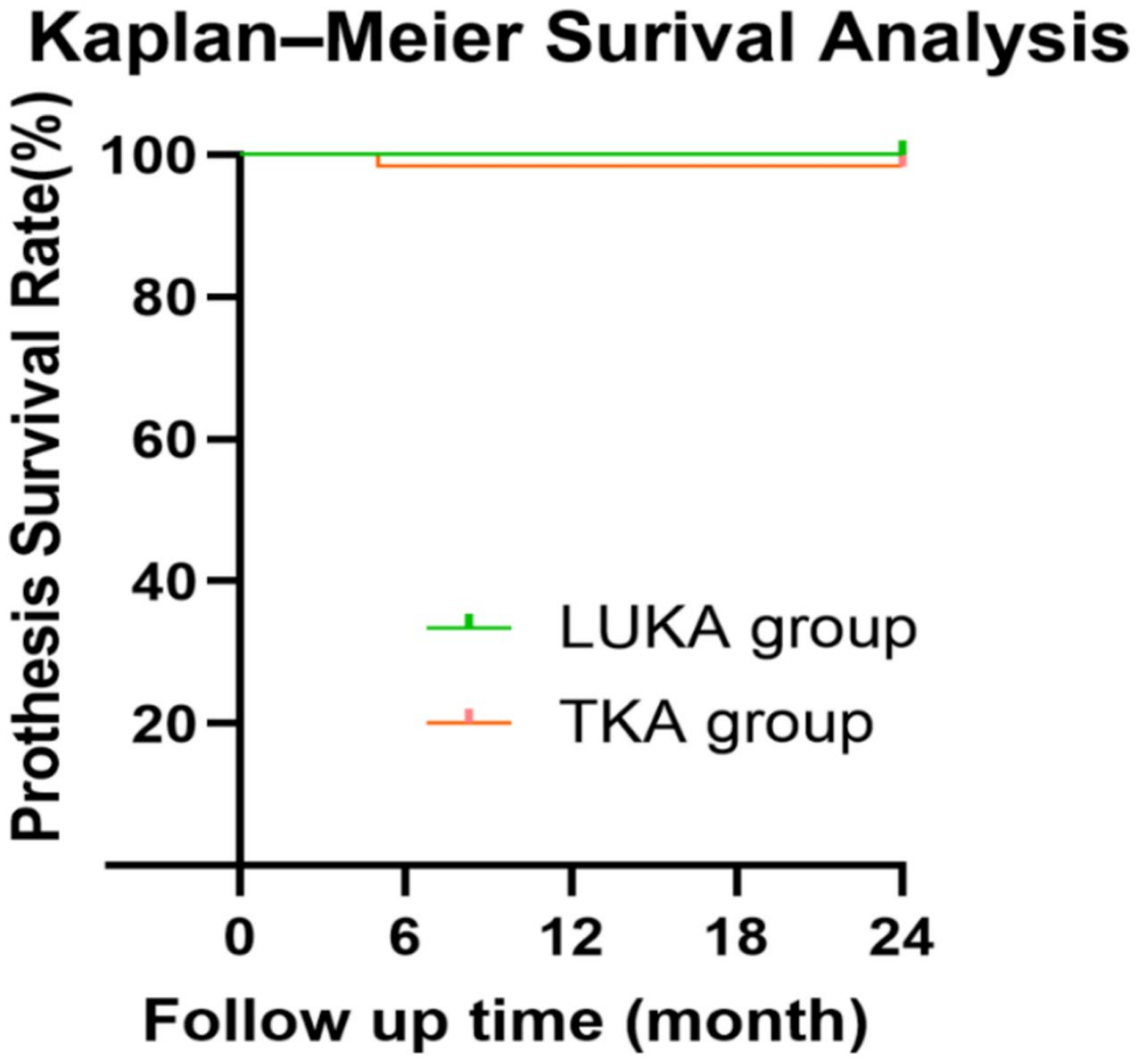
Kaplan–Meier curves for reoperation-free survival after LUKA and TKA through 24 months. Any reoperation on the index knee, including DAIR for PJI, was treated as an event. Curves are presented descriptively given the low event rate.

No revisions occurred in the LUKA group. In the TKA group, one patient (1.6%) underwent debridement, antibiotics, and implant retention (DAIR) for periprosthetic joint infection (PJI). When any reoperation on the index knee was treated as an event, Kaplan–Meier curves were used to summarise reoperation-free survival through 24 months. With only one event recorded, the curves were interpreted descriptively (Figure 1).

### 3.5 Representative cases

**Figure 2:**
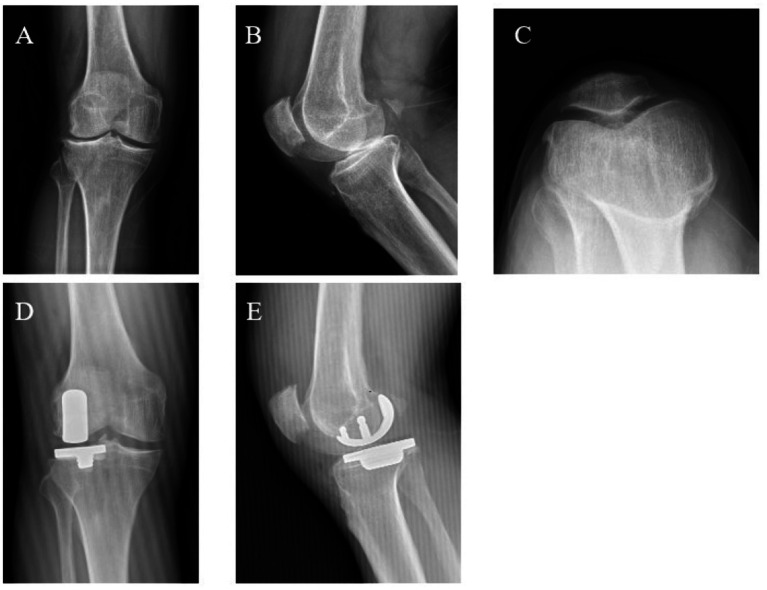
Representative radiographs of a patient treated with LUKA. **(A–C)** Preoperative anteroposterior (AP), lateral, and skyline (axial) views showing LCOA with mild patellofemoral degeneration. **(D,E)** Postoperative AP and lateral views demonstrating no obvious malpositioning on routine radiographs.

**Figure 3:**
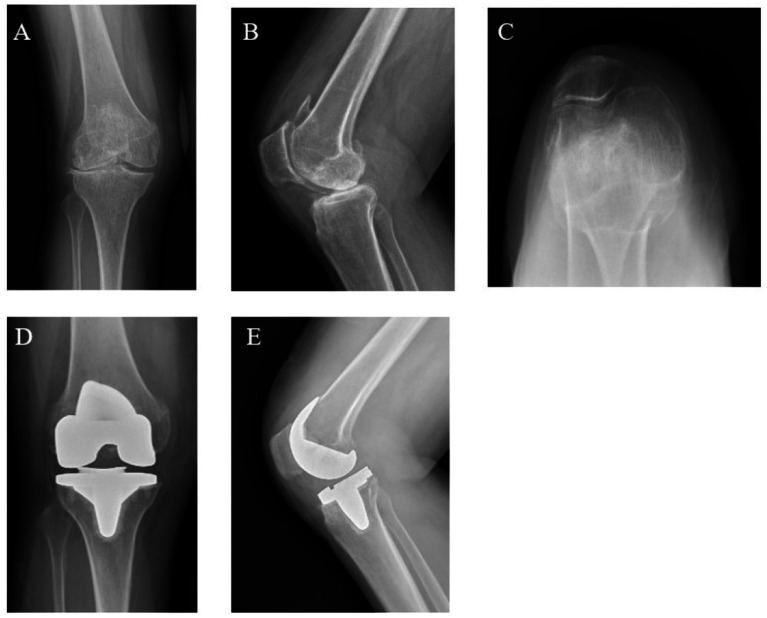
Representative radiographs of a patient treated with TKA. **(A–C)** Preoperative AP, lateral, and skyline (axial) views showing predominant lateral compartment osteoarthritis with preserved medial joint space and mild-to-moderate patellofemoral degeneration. **(D,E)** Postoperative AP and lateral views demonstrating implanted components on follow-up radiographs.

To provide radiographic context, one representative case from each group is shown. In the LUKA case, the KSS improved from 60 preoperatively to 92 at 24 months, and the WOMAC decreased from 65 to 16 (Figure 2). A patient treated with TKA showed a comparable change in KSS and WOMAC over the same follow-up period (Figure 3). These images illustrate typical preoperative findings and implant positioning at follow-up.

## 4 Discussion

In this single-centre cohort of 115 patients with isolated LCOA and coexisting AKP managed through a uniform lateral parapatellar pathway, both LUKA and TKA produced sustained improvements in pain and function over 24 months. LUKA showed an early recovery advantage at 3 months, with higher KSS, lower WOMAC, and less lateral compartment pain, while patellofemoral pain ratings were comparable. By 24 months, no statistically significant between-group differences were detected in functional scores or compartment-specific pain measures; adjusted and propensity-weighted estimates suggested small between-group differences, which should not be interpreted as equivalence in the absence of prespecified margins. By applying the same patellofemoral adjuncts across implant types and reporting compartment-specific pain distribution, the study provides phenotype-focused descriptive evidence rather than a procedure-selection rule.

The role of AKP and PFOA as potential contraindications to UKA remains debated. Kozinn and Scott suggested that exposed subchondral bone in the patellofemoral joint should preclude UKA (23). Later studies, largely from medial UKA cohorts, reported that AKP and medial PFOA were not necessarily associated with worse long-term function or implant survival, whereas severe lateral PFOA, subchondral bone loss, and trochlear erosion were linked to poorer outcomes (24, 25). More recent series have further broadened indications, supporting UKA in selected patients with moderate PFOA (26). Against this background, our cohort indicates that, within a standardised lateral parapatellar pathway incorporating patellar denervation and lateral retinacular release, no clear between-group difference in anterior pain relief was detected through 24 months in patients with isolated LCOA and coexisting AKP.

Prior series of LUKA for isolated LCOA have reported favourable function and survivorship. In the present cohort, AKP may reflect a multifactorial phenotype related to loading, soft-tissue restraint, and patellofemoral morphology rather than a single radiographic lesion (27). With patellar denervation and lateral retinacular release applied in both groups and without patellar resurfacing, LC-VAS and PFC-VAS trajectories were broadly similar between LUKA and TKA through 24 months. This design therefore allows a pragmatic description of early recovery and compartment-specific pain distribution in a phenotype-defined population, while keeping the patellofemoral “co-intervention” constant. Generalisability will depend on local pathways, including surgical approach, patellofemoral management, implant selection, and case mix.

The influence of PFOA on UKA outcomes remains unsettled. A meta-analysis by Wu et al. (28) reported that lateral, but not medial, PFOA was associated with slightly lower postoperative KSS after UKA, whereas trochlear changes showed no consistent association. Gaggiotti et al. (29) likewise found limited relationships between patellofemoral status and clinical outcomes or implant survival, suggesting that PFOA severity should inform, rather than determine, treatment selection. In our LUKA cohort, patients with mild-to-moderate patellofemoral degeneration experienced meaningful symptom improvement, and no between-group difference in patellofemoral pain at follow-up was detected compared with TKA. This pattern is compatible with the hypothesis that lateral compartment unloading, together with standardised patellofemoral adjuncts (including patellar denervation and lateral retinacular release), may contribute to symptom relief. However, without dynamic functional metrics or dedicated patellofemoral imaging and because these adjuncts were applied uniformly, the present study cannot test this hypothesis, disentangle their individual effects or infer specific mechanisms (30–32).

Because patellar denervation and lateral retinacular release were applied in both groups (with release performed when indicated), and patellofemoral imaging/tracking metrics were not systematically collected, the present study cannot isolate the contribution of individual peripatellar adjuncts or support causal inference regarding anterior pain relief. Nevertheless, the overall improvement pattern is broadly consistent with prior studies reporting improvements in AKP after patellar denervation and/or lateral retinacular release in the arthroplasty setting (33–35), and generalisability may depend on local pathways, particularly the surgical approach and patellofemoral management strategy.

Key limitations include the retrospective, single-centre design and the 24-month follow-up window. Treatment allocation was non-random and may be subject to allocation bias and structural selection bias related to eligibility constraints for LUKA versus TKA. Although regression adjustment and propensity score weighting were applied, these approaches address measured covariates only, and residual confounding (including unmeasured patellofemoral morphology, activity level, and patient expectations) cannot be excluded; temporal bias due to evolving practise patterns may also persist. Postoperative radiographic alignment and component positioning were not quantitatively assessed, limiting interpretation of alignment-related influences. At 24 months, LC-VAS/PFC-VAS values were generally low and KSS values were high, suggesting possible floor/ceiling effects; therefore, non-significance should be interpreted as no clear between-group difference within this sample and measurement range. Adjusted estimates were small relative to published MCID thresholds (KSS 5 points; WOMAC total 10 points) (36, 37), but equivalence or causality cannot be inferred. Reoperation events were rare and survivorship analyses were descriptive. Finally, LC-VAS and PFC-VAS are pragmatic localisation ratings without formal psychometric validation, and the use of a single surgeon within a uniform pathway may limit external validity. Multicentre studies with quantitative radiographic assessment, patellofemoral-specific measures, and longer follow-up are warranted.

## 5 Conclusion

Within a standardised lateral parapatellar pathway with patellar denervation and lateral retinacular release, LUKA was associated with faster early recovery and lower lateral compartment pain at 3 months while reducing operative burden. By 24 months, no statistically significant between-group differences were detected in pain distribution (LC-VAS and PFC-VAS) or functional outcomes (KSS and WOMAC). Although this was not an equivalence study, adjusted and propensity score–weighted estimates were small, and the corresponding 95% CIs were generally within published MCID thresholds (KSS 5 points; WOMAC total 10 points), suggesting that large clinically important differences are less likely, without implying equivalence. Larger multicentre studies with longer follow-up remain necessary to confirm generalisability and rare implant-related events.

## Data Availability

The raw data supporting the conclusions of this article will be made available by the authors, without undue reservation.
